# Hypercalcemia Complicated by Rhabdomyolysis and Acute Kidney Injury

**DOI:** 10.7759/cureus.80177

**Published:** 2025-03-06

**Authors:** Kyosuke Omata, Hirotaka Fukasawa, Mai Kaneko, Takashi Matsuyama, Ryuichi Furuya

**Affiliations:** 1 Renal Division, Department of Internal Medicine, Iwata City Hospital, Iwata, JPN

**Keywords:** acute kidney injury, calcification, denosumab, hypercalcemia, rhabdomyolysis

## Abstract

Rhabdomyolysis is a condition resulting from muscle breakdown due to trauma, extreme exertion, or seizures, causing the release of intracellular contents including potassium and phosphate (Pi). Abnormalities in serum calcium (Ca) levels have also been reported in rhabdomyolysis, although its exact mechanism remains controversial. A 57-year-old male was admitted due to tonic-clonic seizures caused by epilepsy. On the second day of hospitalization, he exhibited high creatine phosphokinase (CPK) levels (39,253 U/L) and elevated serum creatinine (Cr) levels (2.31 mg/dL), indicating rhabdomyolysis and acute kidney injury (AKI). Because of anuria and azotemia, he started hemodialysis from the third day. Subsequently, renal function gradually improved, and he was weaned off hemodialysis. However, around the 20th day of hospitalization, serum Ca levels began to rise, reaching a peak of 13.3 mg/dL. A computed tomography (CT) scan showed calcification in the left-side muscles including the pectoralis major and upper arm, and bone scintigraphy also demonstrated a high accumulation of technetium-99m hydroxymethylene diphosphonate (^99m^Tc-HMDP) in the same areas. Treatment with denosumab was administered for hypercalcemia, and serum Ca levels returned to normal. On the 45th day, a follow-up CT scan showed the disappearance of the calcific deposits, and no accumulation was seen in the bone scintigraphy.

## Introduction

Rhabdomyolysis is a condition resulting from muscle breakdown due to trauma, extreme exertion, or seizures, causing the release of intracellular contents including potassium and phosphate (Pi), which can result in acute kidney injury (AKI) [1,2].

In hospitalized patients, hypercalcemia is often caused by malignancy, primary hyperparathyroidism, and vitamin D intoxication [3,4]. On the other hand, abnormalities in serum calcium (Ca) levels have been reported in rhabdomyolysis, although its exact mechanism remains controversial [2,5,6].

Herein, we present a case in which marked hypercalcemia developed during the recovery phase of rhabdomyolysis triggered by seizures. We also report serial findings of the calcific lesions, which could have contributed to hypercalcemia, using computed tomography (CT) scans and bone scintigraphy.

## Case presentation

A 57-year-old male was transferred to our hospital due to recurrent tonic-clonic seizures affecting the left side of the body. He had a medical history of epilepsy and type 2 diabetes mellitus. Consciousness was impaired (Glasgow Coma Scale of E1V1M1) and no obvious external trauma was noted in the body. Physical examination showed height of 175 cm, weight of 108.7 kg, and body mass index (BMI) of 34.5 kg/m^2^. Vital signs were blood pressure of 120/101 mmHg, pulse rate of 155 bpm, body temperature of 40.1 °C, and saturation of percutaneous oxygen (SpO_2_) of 71% (room air). Laboratory studies on admission revealed white blood cell (WBC) count of 22,900/mm^3^, hemoglobin of 14.8 g/dL, serum albumin of 4.0 g/dL, creatine phosphokinase (CPK) level of 133 U/L, blood urea nitrogen (BUN) of 17 mg/dL, serum creatinine (Cr) of 1.51 mg/dL, estimated glomerular filtration rate of 39 mL/min/1.73 m^2^, sodium of 143 mEq/L, potassium of 4.4 mEq/L, corrected Ca of 8.4 mg/dL, Pi of 4.3 mg/dL, and C-reactive protein of 0.06 mg/dL. After he was admitted to the neurology department, he was treated with anticonvulsants on mechanical ventilation.

On the second day of hospitalization, the serum CPK level increased to 39,253 U/L and the serum Cr level increased to 2.23 mg/dL. The serum myoglobin level also increased to 284,000 ng/mL (reference: 0-154.9 ng/mL) and urinary occult blood was positive (3+) without red blood cells on sediment examination. The patient was referred to the nephrology department and was diagnosed with rhabdomyolysis-induced AKI.

**Figure 1:**
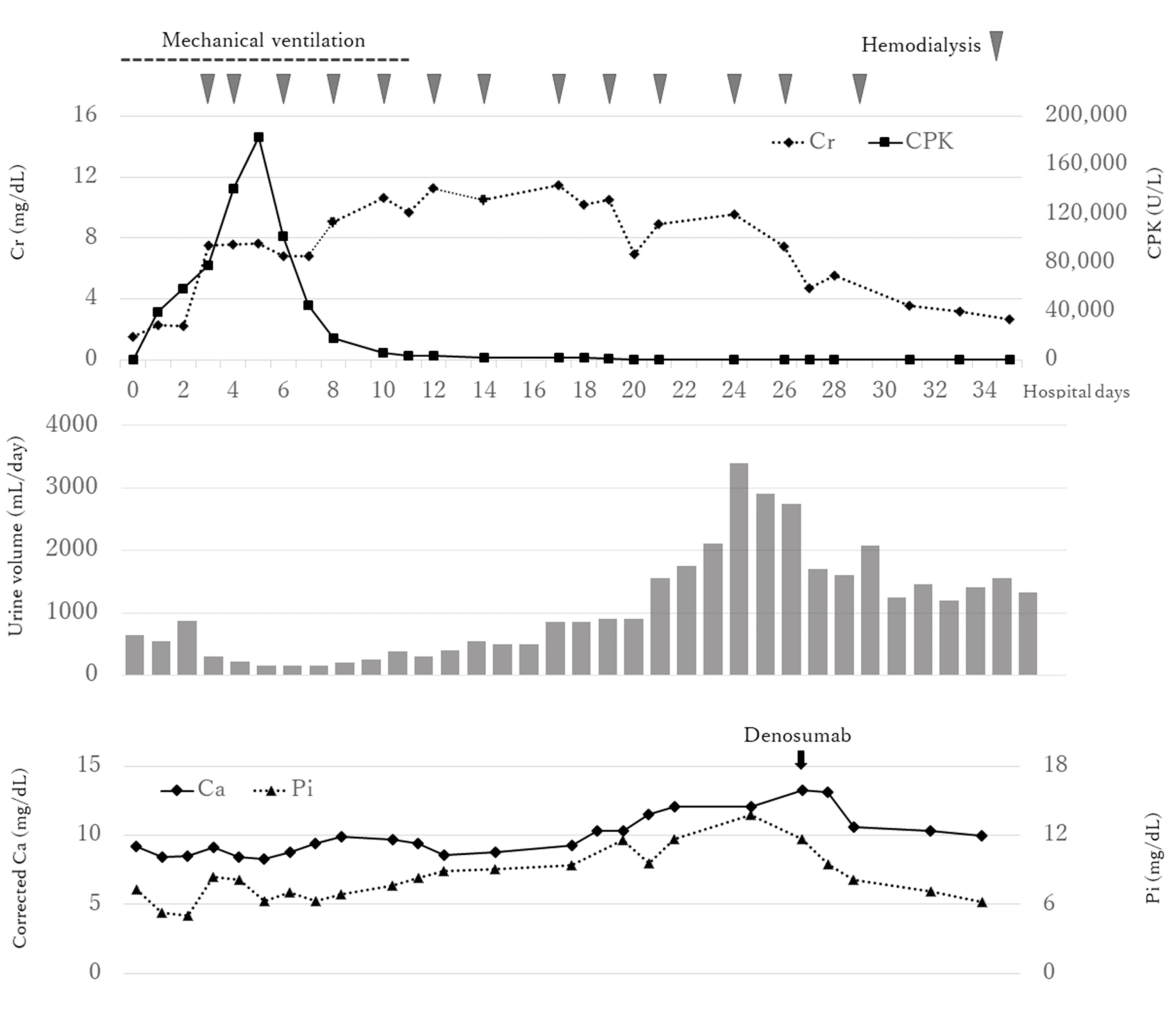
Clinical course after admission Ca, calcium; CPK, creatine phosphokinase; Cr, creatinine; Pi, phosphate.

**Figure 2:**
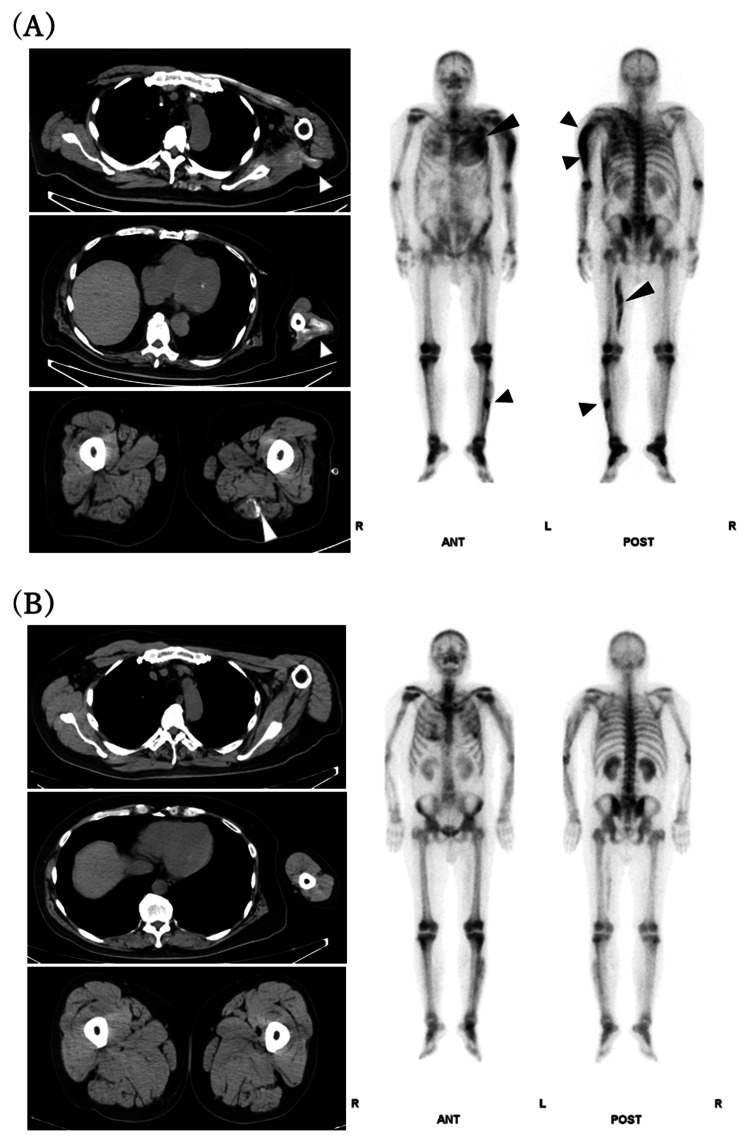
Whole-body plain CT scans and bone scintigraphy (A) At the onset of hypercalcemia (Day 26), calcium deposits (white arrowheads) are seen in the left pectoralis muscle and upper arm, as well as other muscles on the left side. Corresponding high uptake of ^99m^Tc-HMDP is observed in the same sites (black arrowheads). (B) After improvement of hypercalcemia (Day 45), these calcium deposits in the muscles have disappeared, and there is no longer any high uptake of ^99m^Tc-HMDP in those regions. ^99m^Tc-HMDP, technetium-99m hydroxymethylene diphosphonate.

On the third day of hospitalization, the patient was started hemodialysis because he became anuric and his renal function rapidly deteriorated as BUN, serum Cr, and potassium levels increased to 67 mg/dL, 7.54 mg/dL, and 6.8 mEq/L, respectively. The serum CPK level peaked at 182,900 U/L on the sixth day and gradually declined. As rhabdomyolysis improved, urine output gradually increased from around the 20th day, and he was successfully weaned off hemodialysis (Figure 1). Although serum corrected Ca level was mildly low at 8.4 mg/dL on admission, it began to rise as rhabdomyolysis improved, reaching a peak of 13.3 mg/dL on the 26th day (Table 1, Figure 1). Serum corrected Ca levels were calculated using Payne’s formula: corrected Ca level (mg/dL) = measured Ca level (mg/dL) + (4 - serum albumin level [g/dL]) [7]. To investigate the cause of hypercalcemia, a CT scan was performed, and it showed calcification in the left-side muscles including the pectoralis major muscle, triceps brachii muscle, and biceps femoris muscle. Correspondingly, bone scintigraphy revealed a high accumulation of technetium-99m hydroxymethylene diphosphonate (^99m^Tc-HMDP) in these areas (Figure 2A). Secondary hypercalcemia due to malignancy, hyperparathyroidism, and vitamin D toxicity were also ruled out (Table 1). The isotonic electrolyte solution of 1000-2000 mL daily depending on the urine volume was administered to treat hypercalcemia, although hypercalcemia did not improve. On the 26th day of hospitalization, denosumab was administered, and the serum Ca levels returned to normal. A follow-up CT scan on the 45th day showed complete resolution of the calcific lesions, and no accumulation was noted in the bone scintigraphy (Figure 2B). At 15 months before and after hospitalization, his serum corrected Ca and Pi levels maintained within the normal range (9.0 and 2.9 mg/dL before hospitalization, and 9.0 and 2.7 mg/dL after hospitalization, respectively).

**Table 1:** Laboratory findings on the 26th day of hospitalization at the peak of hypercalcemia ACE, angiotensin-converting enzyme; BAP, bone-specific alkaline phosphatase; Ca, calcium; FE, fractional excretion; IGRA, interferon gamma release assay; OH, hydroxy; PTH, parathyroid hormone; PTHrP, parathyroid hormone-related peptide; Pi, phosphate; TRACP, tartrate-resistant acid phosphatase; Vit, vitamin.

Measurement item	Unit	Value	Reference value
Serum Albumin	g/dL	2.6	4.1-5.1
Corrected Ca	mg/dL	13.3	8.8-10.1
Pi	mg/dL	11.4	2.7-4.6
Ionized Ca	mEq/L	2.79	2.41-2.72
Intact PTH	pg/mL	19	10-65
25(OH)Vit D_3_	ng/mL	5	10.0-70.0
1,25(OH)_2_Vit D_3_	pg/mL	9.4	20.0-60.0
ACE	U/L	8.3	8.3-21.4
IGRA		(-)	(-)
PTHrP	pmol/L	<1.0	<1.1
TRACP-5b	mU/dL	422	170-590
BAP	μg/L	26.4	3.7-20.9
FECa	%	31.9	2.0-4.0
FEPi	%	50.5	10.0-20.0

## Discussion

Rhabdomyolysis is a condition in which muscle tissue undergoes necrosis or lysis due to internal or external factors, releasing large amounts of myoglobin and cellular contents into the bloodstream [1]. This can lead to complications such as hyperkalemia, hyperphosphatemia, metabolic acidosis, compartment syndrome, and AKI [2]. Regarding serum Ca levels in rhabdomyolysis, it is known that serum Ca levels often decrease during the acute phase and increase during the recovery phase [5]. This Ca metabolic abnormality has been reported in up to 33% of rhabdomyolysis cases [8]. During the acute phase, Pi released from the damaged muscle cells binds with Ca to form calcium phosphates, leading to Ca deposition into the injured muscle. In the recovery phase, the dissolution of these calcium phosphates into the bloodstream results in hypercalcemia. Hadjis et al. [5] demonstrated this process using CT scans and muscle biopsies. However, the exact mechanisms underlying the release of calcium phosphates during the recovery phase are not fully understood. Several studies suggest that the decrease in serum Pi levels due to renal recovery (diuresis) causes hypercalcemia to maintain the calcium phosphate product (Ca x Pi) [6,9]. On the other hand, Meneghini et al. [10] reported several cases in which hypercalcemia had developed during the oliguric phase of AKI. In this case, hypercalcemia persisted despite continuous hyperphosphatemia, indicating that mechanisms independent of Ca x Pi may be involved. Other proposed mechanisms include secondary hyperparathyroidism following acute hypocalcemia [11] or an increase in active vitamin D during renal recovery [12]. However, in a report by Meneghini et al. [10], which summarized cases of rhabdomyolysis complicated by AKI, 11 cases had elevated intact parathyroid hormone (PTH) levels among 34 cases, and only two cases had elevated 25-hydroxyvitamin D_3_ levels among nine cases. These findings suggest that the Ca metabolism abnormalities in rhabdomyolysis cannot be simply explained by the changes in hormones or vitamins. In our case, both intact PTH and 1,25-dihydroxyvitamin D_3_ levels were low during hypercalcemia, and then the involvement of hyperparathyroidism and the effect of increased vitamin D levels were unlikely.

We also investigated other factors such as malignancy, sarcoidosis, and tuberculosis as possible causes of hypercalcemia, but those factors were ruled out based on the radiological and laboratory findings (Table 1). Moreover, Ca replacement therapy was not administered for the acute-phase hypocalcemia in our case. Although the involvement of immobility could not be completely ruled out, early rehabilitation and the absence of elevated bone resorption markers (TRACP [tartrate-resistant acid phosphatase]-5b) suggested that its effect was minimal [13].

There have been several reports on Ca metabolism abnormalities in rhabdomyolysis, although those reports observed the progression of calcific lesions using CT scans or bone scintigraphy at a single time point [5,6,8,9,14-17]. On the other hand, our case observed the serial changes in calcific lesions using two modalities, CT scans and bone scintigraphy. Furthermore, those calcific lesions were primarily observed on the left side of the body corresponding to the site of seizures. Taken together, we speculated the mechanism of hypercalcemia complicated with rhabdomyolysis in our case as follows: (i) Pi released from the damaged muscle cells bound with Ca to form calcium phosphates, leading to Ca deposition into the injured muscle during the acute phase; and (ii) the dissolution of these calcium phosphates into the bloodstream resulted in hypercalcemia in the recovery phase. Recently, Alkaissi and McFarlane [18] also reported serial radiographical findings in a patient who had been applied Ca sulfate beads at the surgical sites, which developed hypercalcemia probably due to the dissolution from the sites. Furthermore, our case responded well to denosumab, a monoclonal antibody targeting the receptor activator of nuclear factor kappa-B ligand (RANKL), effectively reducing serum Ca levels. In the presence of AKI, denosumab can be a useful treatment option as it does not require renal excretion, although hemodialysis using a dialysis fluid of low calcium concentration may be another treatment option.

From the point of view of hyperphosphatemia, familial hyperphosphatemic tumoral calcinosis (FHTC) could be listed as one of the differential diagnoses of the disease that shows a similar phenotype to our case [19,20]. On the other hand, hyperphosphatemia was transiently observed just in the recovery phase of rhabdomyolysis, hypercalcemia was rarely observed in FHTC, and calcific lesions were observed in the site of injured muscles, but not in the periarticular sites typically found in FHTC. In addition, we could not explain the reason for the suppressed serum 1,25-dihydroxyvitamin D_3_ level, which should be elevated or inappropriately normal for the degree of hyperphosphatemia in FHTC [21].

## Conclusions

We presented a case of hypercalcemia developed in the recovery phase of rhabdomyolysis. To the best of our knowledge, this case is the first report that observed the serial changes of calcific lesions using two modalities, CT scans and bone scintigraphy. Although the exact mechanisms of hypercalcemia in rhabdomyolysis are still controversial, it was speculated that the dissolution of the calcific lesions into the bloodstream resulted in hypercalcemia. In addition, denosumab was found to be effective in normalizing the Ca levels, although denosumab might actually affect bones. This case emphasizes the importance of monitoring Ca levels and suggests a treatment option such as denosumab in patients with hypercalcemia following rhabdomyolysis.

## References

[1] Bosch X, Poch E, Grau JM (2009). Rhabdomyolysis and acute kidney injury. N Engl J Med.

[2] Torres PA, Helmstetter JA, Kaye AM, Kaye AD (2015). Rhabdomyolysis: Pathogenesis, diagnosis, and treatment. Ochsner J.

[3] Mousseaux C, Dupont A, Rafat C (2019). Epidemiology, clinical features, and management of severe hypercalcemia in critically ill patients. Ann Intensive Care.

[4] Walker MD, Shane E (2022). Hypercalcemia: A review. JAMA.

[5] Hadjis T, Grieff M, Lockhat D, Kaye M (1993). Calcium metabolism in acute renal failure due to rhabdomyolysis. Clin Nephrol.

[6] Shrestha SM, Berry JL, Davies M, Ballardie FW (2004). Biphasic hypercalcemia in severe rhabdomyolysis: Serial analysis of PTH and vitamin D metabolites. A case report and literature review. Am J Kidney Dis.

[7] Payne RB, Little AJ, Williams RB, Milner JR (1973). Interpretation of serum calcium in patients with abnormal serum proteins. Br Med J.

[8] Hechanova LA, Sadjadi SA (2014). Severe hypercalcemia complicating recovery of acute kidney injury due to rhabdomyolysis. Am J Case Rep.

[9] Ikechi D, Koga Y, Harada K, Yagi T, Todani M, Fujita M, Tsuruta R (2022). A case of delayed hypercalcemia during diuretic phase of acute kidney injury due to rhabdomyolysis: Evaluation of heterotopic calcification by serial CT. J Jpn Soc Intensive Care Med.

[10] Meneghini LF, Oster JR, Camacho JR, Gkonos PJ, Roos BA (1993). Hypercalcemia in association with acute renal failure and rhabdomyolysis. Case report and literature review. Miner Electrolyte Metab.

[11] Llach F, Felsenfeld AJ, Haussler MR (1981). The pathophysiology of altered calcium metabolism in rhabdomyolysis-induced acute renal failure. Interactions of parathyroid hormone, 25-hydroxycholecalciferol, and 1,25-dihydroxycholecalciferol. N Engl J Med.

[12] Akmal M, Bishop JE, Telfer N, Norman AW, Massry SG (1986). Hypocalcemia and hypercalcemia in patients with rhabdomyolysis with and without acute renal failure. J Clin Endocrinol Metab.

[13] Halleen JM, Tiitinen SL, Ylipahkala H, Fagerlund KM, Väänänen HK (2006). Tartrate-resistant acid phosphatase 5b (TRACP 5b) as a marker of bone resorption. Clin Lab.

[14] Imai R, Akimoto T, Takeda S, Muto S, Kusano E (2014). Is calcium replacement therapy unnecessary for severe hypocalcemia associated with rhabdomyolysis?. Clin Exp Nephrol.

[15] Ding LN, Wang Y, Tian J, Ye LF, Chen S, Wu SM, Shang WB (2019). Primary hypoparathyroidism accompanied by rhabdomyolysis induced by infection: A case report. World J Clin Cases.

[16] Bedani PL, Gilli P (1995). Hypertensive emergency due to hypercalcemia after acute renal failure secondary to rhabdomyolysis. Nephron.

[17] Zakout R, do Carmo M, Freitas P (2013). Reversible myocardial calcification following severe leptospirosis complicated with rhabdomyolysis-induced acute kidney injury and magnesium-wasting nephropathy. J Med Cases.

[18] Alkaissi HR, McFarlane SI (2022). Hypercalcemia in a 67-year-old female following the use of calcium sulfate beads: A case report and review of literature. Cureus.

[19] Ramnitz MS, Gafni RI, Collins MT (2018). Hyperphosphatemic familial tumoral calcinosis. GeneReviews® [Internet].

[20] Chakhtoura M, Ramnitz MS, Khoury N (2018). Hyperphosphatemic familial tumoral calcinosis secondary to fibroblast growth factor 23 (FGF23) mutation: A report of two affected families and review of the literature. Osteoporos Int.

[21] Dauchez A, Souffir C, Quartier P, Baujat G, Briot K, Roux C (2019). Hyperphosphatemic familial tumoral calcinosis with Galnt3 mutation: Transient response to anti-interleukin-1 treatments. JBMR Plus.

